# Novel copper-containing ferrite nanoparticles exert lethality to MRSA by disrupting MRSA cell membrane permeability, depleting intracellular iron ions, and upregulating ROS levels

**DOI:** 10.3389/fmicb.2023.1023036

**Published:** 2023-02-09

**Authors:** Jinhua Ye, Fangpeng Hou, Guanyu Chen, Tianyu Zhong, Junxia Xue, Fangyou Yu, Yi Lai, Yingjie Yang, Dedong Liu, Yuantong Tian, Junyun Huang

**Affiliations:** ^1^Analytical Laboratory of Basic Medical College, Gannan Medical University, Ganzhou, Jiangxi, China; ^2^Center for Immunology, Key Laboratory of Prevention and Treatment of Cardiovascular and Cerebrovascular Diseases, Ministry of Education, Gannan Medical University, Ganzhou, Jiangxi, China; ^3^Department of Clinical Laboratory, The First Affiliated Hospital of Gannan Medical University, Ganzhou, Jiangxi, China; ^4^Department of Chemistry and Biochemistry, Kent State University, Kent, OH, United States; ^5^Department of Clinical Laboratory, Shanghai Pulmonary Hospital of Tongji University, Shanghai, China; ^6^Pharmacology Department, Gannan Medical University, Ganzhou, Jiangxi, China

**Keywords:** MRSA, Cu@Fe NPs, antibacterial mechanism, cell membrane, Fe, ROS

## Abstract

**Objective:**

The widespread use of antibiotics has inevitably led to the emergence of multidrug-resistant bacterial strains, such as methicillin-resistant *Staphylococcus aureus* (MRSA), making treatment of this infection a serious challenge. This study aimed to explore new treatment strategies for MRSA infection.

**Methods:**

The structure of Fe_3_O_4_ NPs with limited antibacterial activity was optimized, and the Fe^2+^ ↔ Fe^3+^ electronic coupling was eliminated by replacing 1/2 Fe^2+^ with Cu^2+^. A new type of copper-containing ferrite nanoparticles (hereinafter referred to as Cu@Fe NPs) that fully retained oxidation–reduction activity was synthesized. First, the ultrastructure of Cu@Fe NPs was examined. Then, antibacterial activity was determined by testing the minimum inhibitory concentration (MIC) and safety for use as an antibiotic agent. Next, the mechanisms underlying the antibacterial effects of Cu@Fe NPs were investigated. Finally, mice models of systemic and localized MRSA infections was established for *in vivo* validation.

**Results:**

It was found that Cu@Fe NPs exhibited excellent antibacterial activity against MRSA with MIC of 1 μg/mL. It effectively inhibited the development of MRSA resistance and disrupted the bacterial biofilms. More importantly, the cell membranes of MRSA exposed to Cu@Fe NPs underwent significant rupture and leakage of the cell contents. Cu@Fe NPs also significantly reduced the iron ions required for bacterial growth and contributed to excessive intracellular accumulation of exogenous reactive oxygen species (ROS). Therefore, these findings may important for its antibacterial effect. Furthermore, Cu@Fe NPs treatment led to a significant reduction in colony forming units within intra-abdominal organs, such as the liver, spleen, kidney, and lung, in mice with systemic MRSA infection, but not for damaged skin in those with localized MRSA infection.

**Conclusion:**

The synthesized nanoparticles has an excellent drug safety profile, confers high resistant to MRSA, and can effectively inhibit the progression of drug resistance. It also has the potential to exert anti-MRSA infection effects systemically *in vivo*. In addition, our study revealed a unique multifaceted antibacterial mode of Cu@Fe NPs: (1) an increase in cell membrane permeability, (2) depletion of Fe ions in cells, (3) generation of ROS in cells. Overall, Cu@Fe NPs may be potential therapeutic agents for MRSA infections.

## Introduction

*Staphylococcus aureus* (SA) is a Gram-positive bacterium that belongs to the genus Staphylococcus, with approximately 0.8 μm in diameter and arranged in “grape bunches” under the microscope (Edwards and Massey, 2011; Gardete and Tomasz, 2014). It can cause purulent infections of the skin and soft tissues, and is also a major cause of pneumonia, endocarditis, bacteraemia, septicaemia, and many other diseases. It can even be life threatening in severe cases (Humphreys, 2012). The widespread use of antibiotics has inevitably led to the emergence of an increasing number of multidrug-resistant bacterial strains. In particular, methicillin-resistant *S. aureus* (MRSA) is highly pathogenic and a major cause of hospital and community-acquired infections. The prevalence of MRSA infection is gradually increasing in many parts of the world (Blaskovich et al., 2018). MRSA infection is associated with increased mortality, morbidity, and length of hospital stay compared with methicillin-sensitive *S. aureus* (MSSA) infection (Hu et al., 2019; Cheung et al., 2021). MRSA is highly resistant not only to methicillin but also to all other β-lactam and cephalosporin antibiotics with similar structure to methicillin (Vestergaard et al., 2019; Cheung et al., 2021). Vancomycin, which is recognized as the last line of defense against Gram-positive coccal infections and is currently the “gold standard” treatment for MRSA infections, has a relatively high bactericidal activity *in vivo* (Okano et al., 2017). To date, no drug has shown superiority over vancomycin for the treatment of MRSA infections (Holmes et al., 2015; Vestergaard et al., 2019). However, vancomycin exhibits a clear dose–response/dose-toxicity relationship *in vivo*, with an increased risk of nephrotoxicity and ototoxicity if inappropriate doses or prolonged treatment are used (Antonoplis et al., 2018; Czuban et al., 2018). Staphylococcal strains acquire resistance by modifying the terminal dipeptide of the bacterial cell wall peptidoglycan chain from D-alanine-D-alanine (D-Ala-DAla) to D-alanine-D-lactic acid (D-Ala-D-Lac) that reduces the affinity of the dipeptide for vancomycin and preventing disruption of the peptidoglycan cross-links (Okano et al., 2017). What more worrying is that vancomycin-intermediate *S. aureus* (VISA) and vancomycin-resistant *S. aureus* (VRSA) are currently present in some parts of the world (Lakhundi and Zhang, 2018).

Recent researches have suggested that nanoparticles (NPs), particularly metal NPs, open a new avenue for improving antimicrobial efficacy and reducing the incidence of drug resistance, which has attracted our attention. NPs consisting of metals such as gold (Au), silver (Ag), copper (Cu), zinc (Zn), and iron (Fe) have shown bactericidal activity against a wide range of microorganisms. Compared to conventional metal ion formulations, metal NPs are smaller in size and have a larger surface area, allowing them to better adhere to bacterial cell surfaces. Consequently, more metal ions penetrate bacterial cell walls and cell membranes, catalyze the production of reactive oxygen species (ROS), disrupt cell membrane structures and bind to intracellular DNA to realize bactericidal effects (Vimbela et al., 2017). For example, the basic mechanisms underlying the bactericidal action of ZnO NPs include the physical contact of NPs with the bacterial cell wall, production of ROS, and release of free radicals and Zn^2+^ ions (Gharpure and Ankamwar, 2020). Other researchers have found that treatment of *Escherichia coli* (E. coli) and SA with cationic particles of nano-silver can increase the permeability of bacterial cell membranes compared to untreated bacteria, resulting in accelerated leakage of intracellular components (e.g., amino acids, polyamines and organic acids) and disruption of membrane-bound genomic DNA and a significant increase in DNA degradability (Anuj et al., 2019a,b). All these effects on the bacterial membrane eventually induce bacterial death.

Iron oxide nanoparticles (Fe_3_O_4_ NPs) have been approved by the US Food and Drug Administration (FDA) for their clinical use in the treatment of iron deficiency anemia and as MRI contrast agents.(Trujillo-Alonso et al., 2019) Fe_3_O_4_ NPs have been shown to exhibit antibacterial properties. However, the antibacterial effect of Fe_3_O_4_ NPs alone is limited under normal physiological conditions. The oxidative activity of Fe^2+^ is inhibited due to the electronic coupling of Fe^2+^ ↔ Fe^3+^ in their structure. Therefore, in this study, the structure of Fe_3_O_4_ NPs was optimized and a novel copper-containing ferrite nanoparticles (Cu@Fe NPs) was synthesized. The oxidation–reduction activity was fully preserved. This study found, for the first time, that Cu@Fe NPs had significant antibacterial activity against MRSA and could effectively attenuate the progression of drug-resistant bacteria. The antibacterial mechanism of Cu@Fe NPs was further explored based on the drug safety profile. Interestingly, unlike the antibacterial targets of conventional antibiotics, Cu@Fe NPs have a unique mode of antibacterial action. Furthermore, the therapeutic efficacy of the nanoparticles was verified by establishing MRSA systemic and localized infection mouse models. Overall, the novel Cu@Fe NPs synthesized in this study can serve as a valuable lead candidate against MRSA infections.

## Results

### Characterization of Cu@Fe NPs

**Figure 1:**
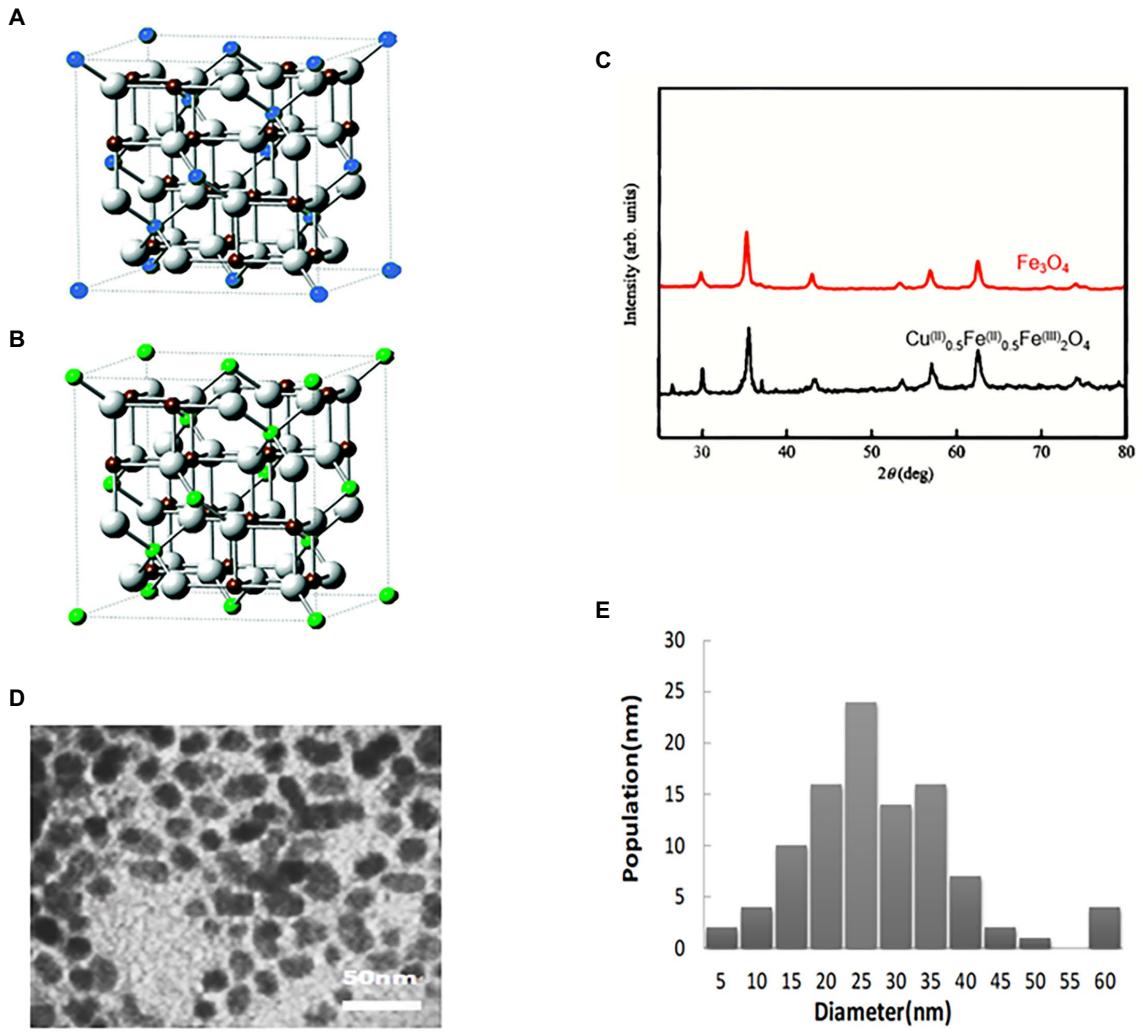
Characterization of Cu@Fe NPs. **(A**,**B)** 3D structures of Fe_3_O_4_ NPs and Cu@Fe_3_ O_4_ NPs. Blue sphere: Fe^2+^; Green sphere: Cu^2+^ /Fe^2+^; Brown sphere: Fe^3+^; White sphere: O. **(C)** X-ray diffraction analysis (XRD) of Cu@Fe NPs. **(D)** Transmission electron microscopy (TEM) of Cu@Fe NPs. **(E)** Dynamic light scattering particle size (DLS) of Cu@Fe NPs.

Initially, the electronic coupling Fe^2+^ ↔ Fe^3+^ in Fe_3_O_4_ NPs was eliminated by replacing 1/2 of the Fe^2+^ in Fe_3_O_4_ NPs with Cu^2+^. The final ratio of Cu^2+^:Fe^2+^:Fe^3+^ substances was 1:1:4, resulting in the synthesis of novel Cu@Fe NPs containing copper ferrite nanoparticles, as shown in the structural model diagram (Figures 1A,B). Its structural formula was Cu^(II)^_x_Fe^(II)^_1-x_0.5Fe^(III)^_2_O_4_ (1 ≥ *X* ≥ 0.5). X-ray diffraction analysis revealed that Cu@Fe NPs were nanoparticles with a single and high-purity physical phase component and no other impurity physical phase components (Figure 1C). The microscopic structure of Cu@Fe NPs was determined using a transmission electron microscope (TEM) (Figure 1D). In addition, the particle size of Cu@Fe NPs was homogeneous and averaged in the range of 10–60 nm by dynamic light scattering (DLS) (Figure 1E).

### Cu@Fe NPs have an excellent drug safety profile and are effective in attenuating the progression of bacterial resistance

**Figure 2:**
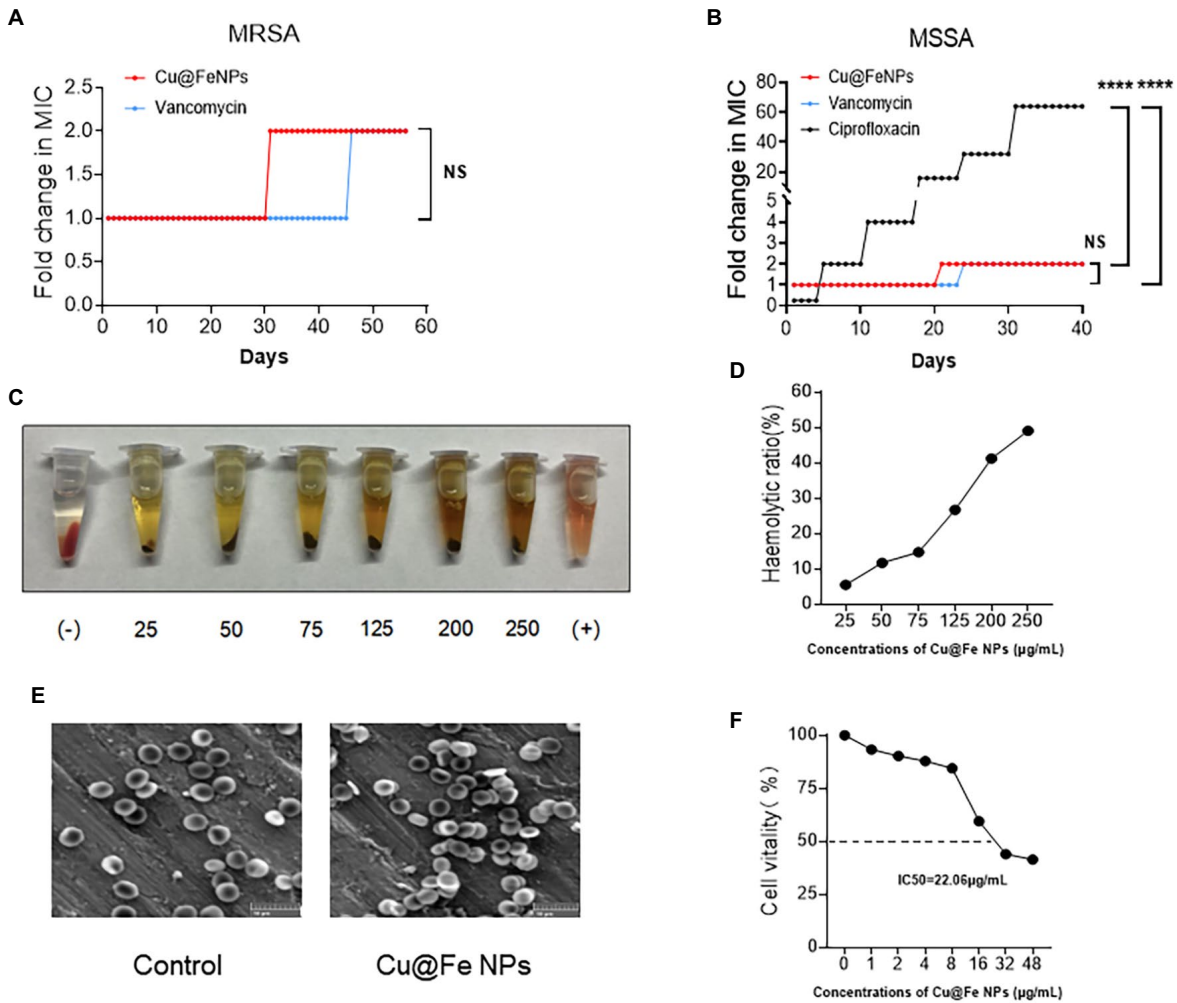
Cu@Fe NPs have excellent antibacterial effect and drug safety. **(A**,**B)** Determination of MIC resistance of Cu@Fe NPs to MSSA and MRSA in successive passages. **(C)** Representative image of the haemolytic response of mRBCs to different concentrations (0–250 μg/mL) of Cu@Fe NPs. **(D)** The haemolytic ratio of mRBCs. **(E)** Typical SEM images of mRBCs treated with PBS or 3.84 μg/mL Cu@Fe NPs. Scale bar, 10 μm (200×). **(F)** Percentage changes in the cell viability of HSF-1 against different concentrations (0–48 μg/mL) of Cu@Fe NPs. **(A**,**B)** Two-way ANOVA with Holm-Sidak’s multiple comparisons test. Data are expressed as mean ± SEM. *NS* versus Vancomycin group. ****p < 0.0001 Ciprofloxacin group.

Accurate determination of bacterial minimum inhibitory concentration (MIC) is important in determining the susceptibility of bacteria to drugs (Grupper et al., 2016). It was found that Cu@Fe NPs had a MIC of 1 μg/mL against MSSA and MRSA, which was comparable to vancomycin. The antibacterial effects of Cu@Fe NPs against VISA were also demonstrated (MIC = 1 μg/mL). After calculation of MIC values, SA resistance tests were performed on Cu@Fe NPs. Cu@Fe NPs showed only a slight increase in MIC from 1 μg/mL to 2 μg/mL for MRSA in the 31st generation compared with the positive control drugs for MRSA and MSSA (vancomycin and ciprofloxacin). MSSA was not highly resistant to vancomycin and Cu@Fe NPs compared with ciprofloxacin. Overall, the MIC value of Cu@Fe NPs remained relatively stable during successive bacterial passages and did not change significantly. It could effectively attenuate the progression of MSSA and MRSA to drug-resistant bacteria (Figures 2A,B). The safety of the drug was explored to ensure that it did not cause major side effects in humans. The drug safety was assessed based on the haemolytic effect of Cu@Fe NPs on mouse red blood cells (mRBCs) and cytotoxicity of human dermal fibroblasts (HSF-1). Cu@Fe NPs were able to dissolve 50% of mouse erythrocytes (HC50) at a concentration of 250 μg/mL (Figures 2C,D). The HC50 far exceeded the previously measured antibacterial therapeutic concentration of Cu@Fe NPs (1 μg/mL), which was 250 times the MIC. Hence, this is a relatively safe therapeutic concentration. Scanning electron microscopy (SEM) results showed that treatment with 3.84 μg/mL Cu@Fe NPs did not alter the normal biconcave disk-like shape of mRBCs compared to the control (Figure 2E). Moreover, the 50% inhibitory concentration (IC50) of Cu@Fe NPs against HSF-1 was 22.06 μg/mL (Figure 2F). The selectivity index (SI) was calculated based on HC50 (for mRBCs) or IC50 (for HSF-1) divided by the MIC of MRSA (SI = HC50/MIC or IC50/MIC). Thus, the selectivity index (SI) values of Cu@Fe NPs for MRSA were 250 (for mRBCs) and 22.06 (for HSF-1). Notably, the IC50 of 22.06 μg/mL was lower than the HC50 concentration of 250 μg/mL, which might be attributed to different sensitivities of cells and tissues to Cu@Fe NPs. However, 22.06 μg/mL far exceeded the MIC concentration of 1 μg/mL, which played an antibacterial role. These findings imply that the synthesized Cu@Fe NPs can effectively avoid bacterial progression toward drug resistance and exhibit a good drug safety profile.

### Cu@Fe NPs pose effective antibacterial activity

**Figure 3:**
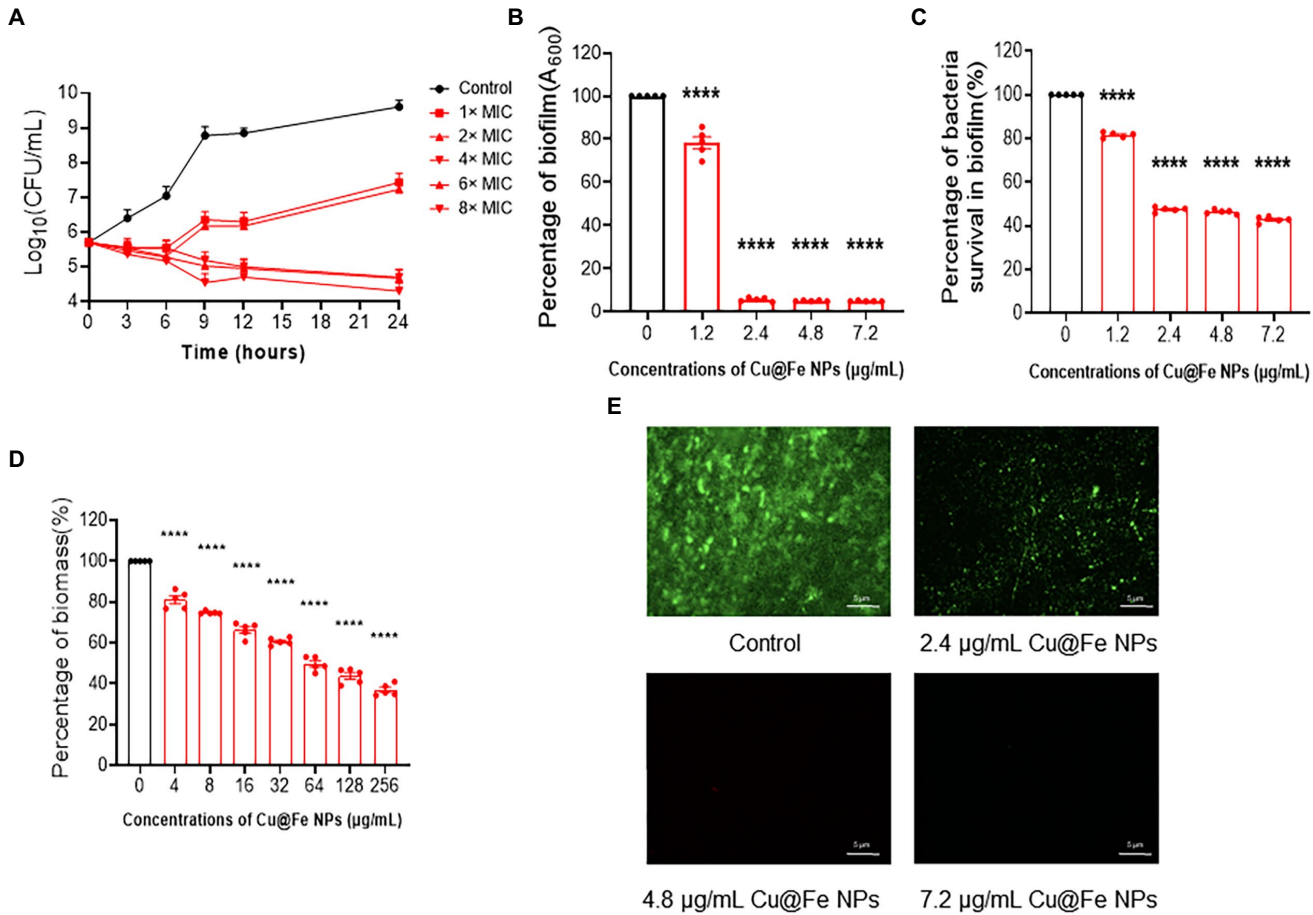
Cu@Fe NPs have good pharmacokinetic properties, and improve the sensitivity of drugs to MRSA by affecting the integrity of biofilms. **(A)** Time-dependent killing effect of different concentrations (0–8 μg/mL) of Cu@Fe NPs on MRSA. **(B**,**C)** Changes in the mass of biofilms formed by MRSA and the percentage of viable bacteria in the biofilms treated with different concentrations (0–7.2 μg/mL) of Cu@Fe NPs. **(D)** Damaging effect of different concentrations (0–256 μg/mL) Cu@Fe NPs on the disruption of MRSA-formed biofilms. **(E)** SYTO9/PI fluorescence staining plots of MRSA biofilms treated with different concentrations (0–7.2 μg/mL) of Cu@Fe NPs. Magnification, 200×. Scale bar, 5 μm. **(A–D)** *n* = 5. Data are from three independent experiments. **(B–D)** One-way ANOVA with Dunnett’s multiple comparisons test. Data are expressed as mean ± SEM. ****p < 0.0001 versus Control group.

Cu@Fe NPs have been identified as an effective anti-MRSA candidate. Next, the kinetics of the antibacterial activity of Cu@Fe NPs were assessed by time-kill analysis of MRSA. It was found that the bacterial solution control showed a gradual increase in the total number of viable bacteria with increasing assay time and rapid bacterial proliferation. However, Cu@Fe NPs showed significant bacterial inhibitory activity at a dose of 4 μg/mL, with a dose-dependent decrease in the number of bacteria and a significant killing effect of Cu@Fe NPs (Figure 3A). Our study showed that in addition to its killing effect on MRSA in the planktonic phase, Cu@Fe NPs significantly inhibited MRSA biofilm formation at a dose of 1.2 μg/mL. This difference became more pronounced when the concentration was further increased (Figures 3B, C). Additionally, a dose of 4 μg/mL was required to eliminate the bacterial biofilms. With increasing concentrations, Cu@Fe NPs became increasingly damaging to MRSA biofilms in a concentration-dependent manner. At a dose of 256 μg/mL, only 40% of the MRSA biofilms remained present (Figure 3D). Fluorescence staining analysis revealed that the green fluorescence intensity of MRSA was 2.4 μg/mL. Treatment with Cu@Fe NPs significantly reduced the fluorescence intensity of MRSA compared to the intact biofilms of control MRSA, particularly at 4.8 and 7.2 μg/mL, indicating that the integrity of MRSA biofilms was severely disrupted (Figure 3E). Cu@Fe NPs exhibited effective anti-biofilm activity, which was more effective in reducing biofilms than vancomycin.

### Cu@Fe NPs demonstrate antibacterial action against MRSA by disrupting cell membrane permeability, depleting intracellular iron ions and upregulating intracellular ROS levels

**Figure 4:**
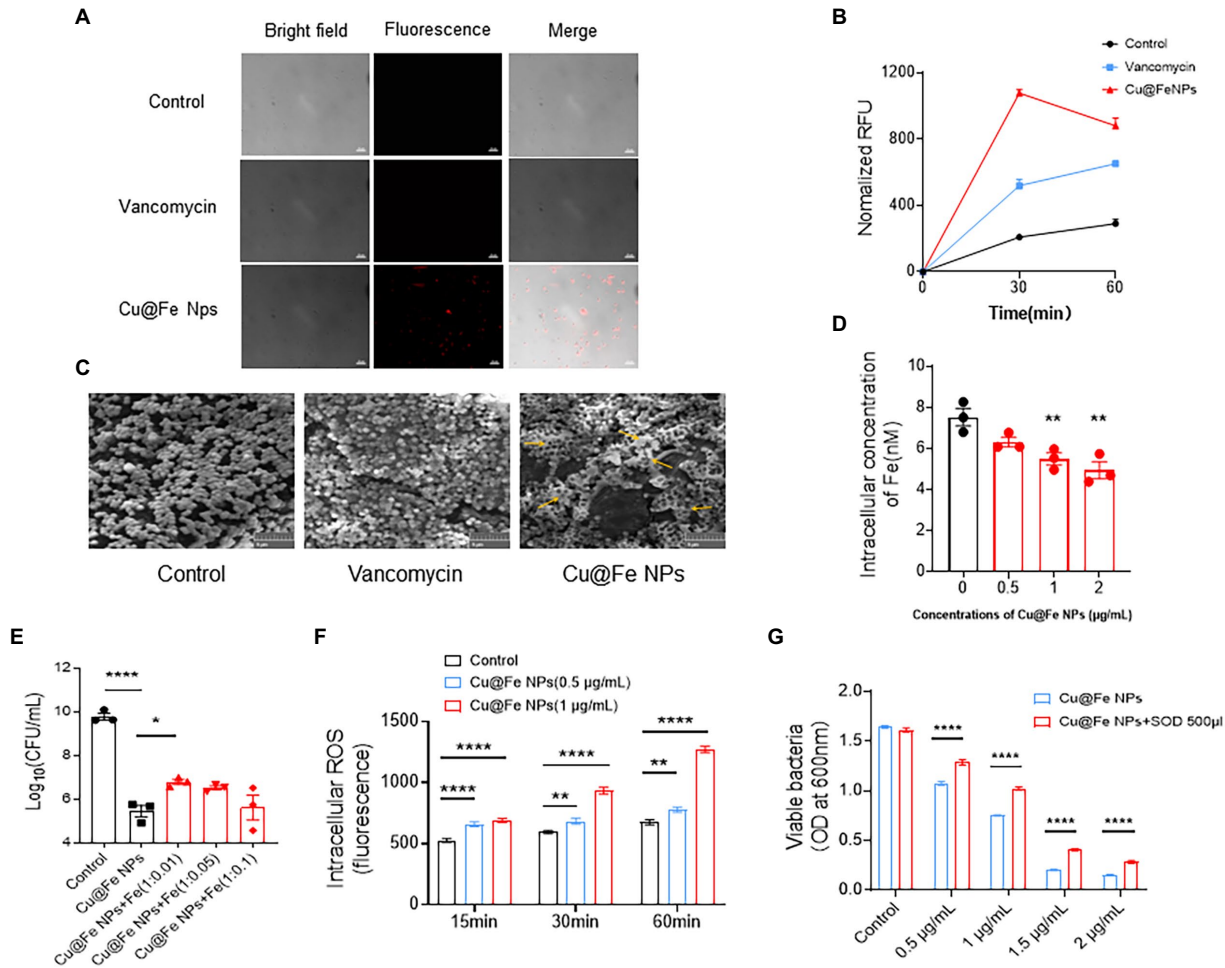
Unique antibacterial mechanisms of Cu@Fe NPs, including the effects on MRSA cell membranes, intracellular iron, and ROS production. **(A**,**B)** Typical microscopic images of PI and changes in the PI fluorescence intensities of MRSA treated with Cu@Fe NPs and vancomycin (3.84 μg/mL for both) over 2 h. Differential interference contrast microscopy (DIC) images (left), fluorescence images (middle) and merged images (right). Magnification, 1,000×. Scale bar, 10 μm. **(C)** Typical SEM images of MRSA treated with Cu@Fe NPs and vancomycin (3.84 μg/mL for both) at 2 h. Scale bar, 5 μm (200×). **(D)** Intracellular iron concentrations of MRSA after treatment with different concentrations (0–2 μg/mL) of Cu@Fe NPs. **(E)** Intracellular iron concentrations of MRSA after co-treatment with Cu@Fe NPs and iron citrate mixture co-treated with ferric citrate. **(F)** Intracellular ROS levels in MRSA bacteria after treatment with different concentrations (0–2 μg/mL) of Cu@Fe NPs. **(G)** Rescue effect of SOD on MRSA-mediated killing by different concentrations (0–1 μg/mL) of Cu@Fe NPs. **(D**–**G)** Data are from three independent experiments. **(D**,**E)** *n* = 3. One-way ANOVA with Dunnett’s multiple comparisons test. **(F**,**G)** *n* = 5. Two-way ANOVA with Holm-Sidak’s multiple comparisons test. Data are expressed as mean ± SEM. *p < 0.05; **p < 0.01; and ****p < 0.0001 compared to controls.

Bacterial membrane permeability was assessed by quantitative uptake of a membrane-impermeable dye, propidium iodide (PI), and the sustained action of Cu@Fe NPs on MRSA was compared to vancomycin treatment at the same concentration. Based on the results of PI fluorescence and quantitative analysis, the addition of 3.84 μg/mL Cu@Fe NPs to MRSA significantly increased the uptake of PI dye more than the effect of vancomycin-treated MRSA on PI uptake at the same concentration (Figures 4A,B). The membrane permeability of MRSA cells treated with Cu@Fe NPs was increased compared with those treated with vancomycin, indicating that Cu@Fe NPs have certain membrane solubilization ability. The morphological structure of the cell surface was observed using SEM. Microscopically, a typical grape-like appearance of MRSA was observed in both control and vancomycin groups, with spherical or slightly oval shapes, neat edges, and smooth surfaces. In contrast, as shown by the orange arrows in Figure 4C, Cu@Fe NPs group showed reduced bacterial numbers, changes in bacterial morphology, adhesion of bacteria to each other in clusters, and leakage of cell contents. In fact, this result is consistent with the known antibacterial mode of action of vancomycin, which exerts its antibacterial activity by inhibiting cell wall synthesis through binding to the D-Ala-D-Ala terminus of the peptidoglycan precursor on the bacterial cell wall, with relatively little effect on membrane permeation (Blaskovich et al., 2018; Stogios and Savchenko, 2020). In contrast to the antibacterial mode of vancomycin, exposure of MRSA to Cu@Fe NPs was associated with significant rupture of the cell membrane.

Considering the key role of iron metabolism in bacterial survival and the preserved Fe^2+^ oxidative activity of Cu@Fe NPs, we investigated whether the antibacterial effect is associated with altered intracellular iron availability in MRSA. Compared to the control, the total intracellular iron level within MRSA continuously decreased in a gradient with increasing concentrations of Cu@Fe NPs (Figure 4D). This indicates that the nanoparticles has a more pronounced iron-depletion effect. To evaluate whether Cu@Fe NPs-induced intracellular iron binding was functionally related to bacterial killing, quantitative viable colony forming units (CFU) of Cu@Fe NPs-treated MRSA were assessed by supplementing with excess free Fe. Compared to the MRSA control treated with Cu@Fe NPs, the number of surviving bacteria increased after supplementation with a certain amount of free Fe. However, this phenomenon was not dose-dependent, and the increase in bacterial numbers was not significant in the Cu@Fe NPs + Fe (1:0.05) and (1:0.1) treatment groups (Figure 4E). This may be due to the existence of an optimal concentration range for exogenous Fe supplementation in bacteria, and excessive Fe supplementation was instead detrimental to the survival of MRSA. Thus, the co-treatment of proper concentrations of Fe with Cu@Fe NPs could rescue the viability of MRSA partially. A lack of iron supply within the bacteria induced by Cu@Fe NPs may trigger a shift from a sequestered state to a planktonic state, whereby the organisms are more sensitive to antibiotics or drugs.

Besides, we observed that MRSA treated with 0.5 μg/mL Cu@Fe NPs for 15 min exhibited a significant increase in ROS levels in a concentration-and time-dependent manner. After 60 min of treatment with 1 μg/mL Cu@Fe NPs, the ROS concentration within the bacteria was twice as high as that of the approaching control (Figure 4F). Normally, endogenous ROS produced by the electron transport chain of aerobic respiration could maintain the normal physiological activity of bacteria, whereas excessive accumulation of exogenous ROS produced by different treatments (e.g., antibiotics) could induce oxidative stress in bacteria, leading to oxidative damage and even affecting their activity. Superoxide dismutase (SOD) is an important member of the family of antioxidant enzymes in biological systems that can maintain cell survival by scavenging intracellular production of catalytic superoxide anion radicals for conversion to hydrogen peroxide and molecular oxygen. After the addition of 500 μL SOD solution, the overall number of bacteria showed a significant decrease as the concentration of Cu@Fe NPs increased, and the number of bacteria treated with the same concentration of Cu@Fe NPs increased significantly compared to the control group without SOD. The results indicated that SOD partially rescued the bacteria viability (Figure 4G).

In summary, our results suggest that Cu@Fe NPs, together with their ability to disrupt bacterial cell membranes, can exhibit antibacterial activity by binding to intracellular iron and upregulating ROS levels in MRSA.

### Establishment of MRSA infection models for *in vivo* validation

**Figure 5:**
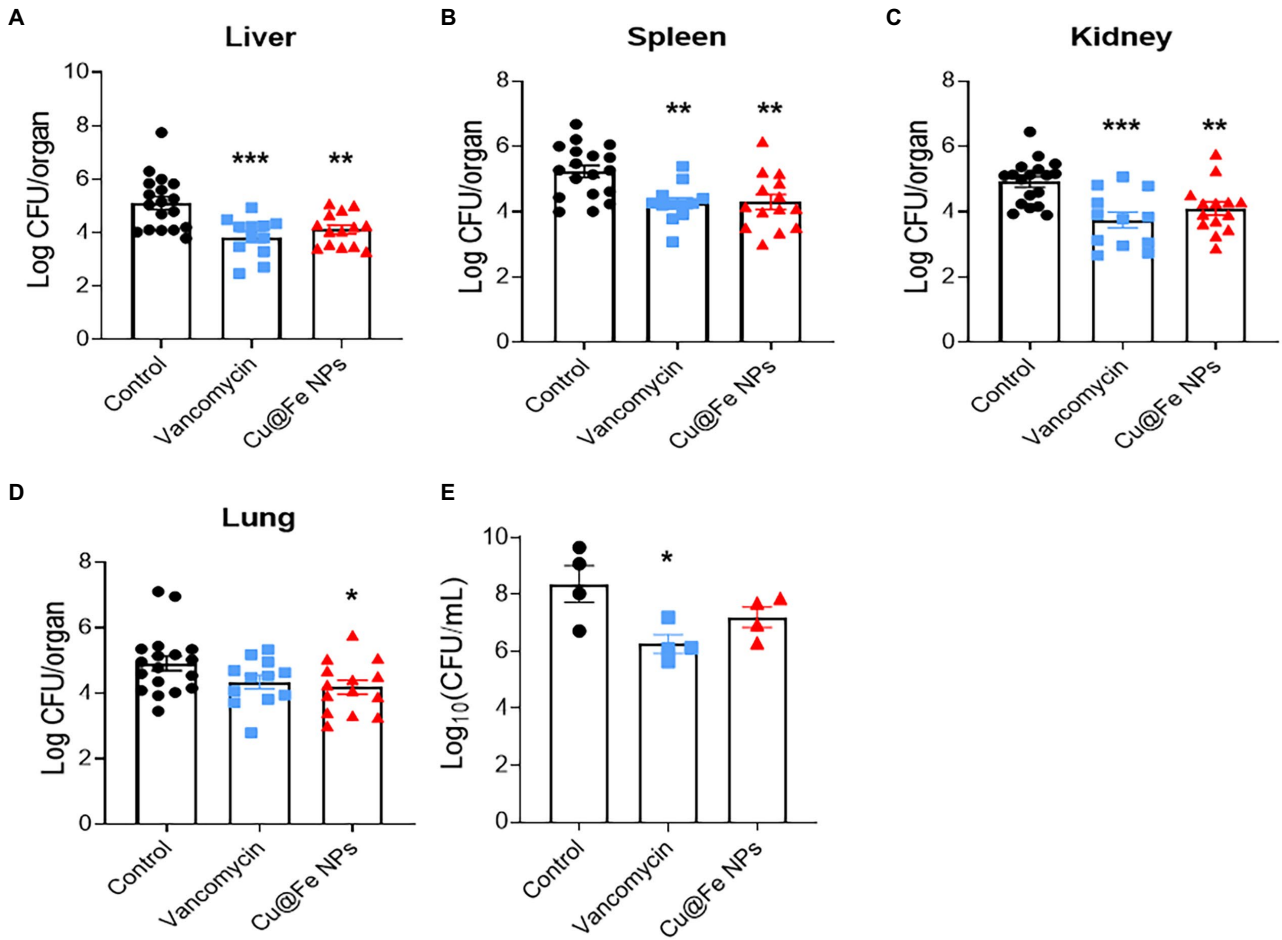
*In vivo* validation for the antibacterial effects of Cu@Fe NPs on MRSA-induced systemic infection and skin wound infection models. **(A–D)** Effects of treatment with 7.8 mg/kg Cu@Fe NPs on the bacterial burden in the liver **(A)**, spleen **(B)**, kidney **(C)**, and lung **(D)** of MRSA-infected mice, respectively. There was a significant decrease in organ CFU in the mice treated with vancomycin or Cu@Fe NPs compared to the untreated group. Control and Cu@Fe NPs groups, *n* = 10. Vancomycin group, *n* = 8. **(E)** CFU status of the infected skin flaps in mice, *n* = 4. CFU counts for the homogenized tissue collected from each mouse were performed in replicates. **(A–D)** Control, *n* = 18. Vancomycin, *n* = 12. Cu@Fe NPs, *n* = 14. Data are from three independent experiments. **(E)** *n* = 4. Data are from two independent experiments. **(A–E)** One-way ANOVA with Dunnett’s mulitple comparisons test. Data are expressed as mean ± SEM versus controls group, *p < 0.05; **p < 0.01; and ***p < 0.001.

The effectiveness of Cu@Fe NPs as antibacterial agents was verified by establishing animal models of MRSA-induced nonlethal systemic and localized infections. The MRSA solution (1 × 10^8^ CFU) was injected intraperitoneally into mice on day 0. On day 1 post-infection, mice were intraperitoneally injected with a single dose of sterile saline, 7.8 mg/kg vancomycin, and 7.8 mg/kg Cu@Fe NPs. Compared to the solvent control group, the mice injected with vancomycin and Cu@Fe NPs showed a significant reduction in intraperitoneal CFU in all organs and tissues, including the liver, spleen, kidney and lung (Figures 5A–D). For local wound infection experiments, a 6-mm full-thickness circular wound was created on the dorsal skin of mice through skin debridement. The MRSA solution (1 × 10^7^ CFU) was inoculated onto the injured skin. The skin wound was immediately covered with a clear semi-permeable Tegaderm-3 M dressing. On day 1 after infection, sterile solvent, vancomycin (0.4 mg/kg), or Cu@Fe NPs (0.4 mg/kg) was administered topically to the infected area of the wound. However, Cu@Fe NPs had no significant effect against skin infection, and vancomycin only had a limited effect (Figure 5E). These results imply that Cu@Fe NPs has a promising antibacterial effect on systemic MRSA infections *in vivo*, but not on localized skin infections.

## Discussion

The misuse of conventional antibiotics has led to the emergence of multidrug-resistant bacteria that have evaded natural antibiotics, or even changed their chemically modified forms, which are commonly known as “superbugs.” One such species is MRSA, a Gram-positive bacterium that poses a serious threat to global health and is particularly difficult to treat (Gupta et al., 2018). “Superbug” infections are often associated with significant complications (Hassuna et al., 2020). The “superbug” and antibiotic resistance are related to approximately 50% of all healthcare-related deaths that is a major complication (Kotb et al., 2018). Therefore, in addition to conventional antibiotic-based treatments, there is an urgent need to develop and synthesize new drugs to counter the damage caused by these superbugs. In 2017, the World Health Organization (WHO) has strongly advocated for the priority development of new drugs for high-risk antimicrobial-resistant pathogens such as *S. aureus* and *S. enteritidis* (Theuretzbacher and Piddock, 2019). Surprisingly, NPs exhibit significant bactericidal activity owing to their unique properties. More importantly, to date, bacteria have not developed resistance to disruption of metal ion homeostasis. Thus, antimicrobial nanomaterials offer a promising alternative strategy to address the global challenge of cellular bacterial infections due to worsening drug resistance.

In the present study, we modified the structure of Fe_3_O_4_ NPs and synthesized a novel nanoparticles. The safety of the drug was ensured. The *in vitro* results showed that Cu@Fe NPs exhibited strong anti-MRSA activity and improved resistance to MRSA. Bacterial biofilms are extracellular composite structures composed of different populations of microorganisms attached to the surface of a substrate, with the microorganisms inside surrounded by a highly hydrated extracellular polymer matrix produced by themselves as a protective way for bacteria to survive and adapt to their surroundings (Saxena et al., 2019). The primary role of biofilm formation during infection is to protect bacteria from phagocytosis, and the bacteria in biofilms are more resistant to antimicrobial agents than the planktonic bacteria (Thurlow et al., 2011). Bacteria under different stresses make biofilms to protect themselves, which in turn leads to prolonged therapeutic intervention and increased resistance to drugs. Cu@Fe NPs can significantly inhibit biofilm formation or remove the formed biofilms, prompting the bacteria to become more sensitive to the drug.

Next, the unique antibacterial mechanisms of Cu@Fe NPs, different from that of conventional antibiotics, were further revealed, which included cell membrane damage, intracellular iron depletion, and upregulation of intracellular ROS levels. Bacterial membranes are rich in enzyme systems and have many important metabolic functions. They provide a relatively stable internal environment for bacterial life activities, function as a barrier for selective substance transport, and exhibit other biological functions such as hormone activity, enzyme reactions, cell recognition and electron transfer (Strahl and Errington, 2017). Although the mechanism of the structural interaction between Cu@Fe NPs and MRSA bacterial membranes has not been elucidated, it is undeniable that Cu@Fe NPs can cause irreversible damage to cell membranes, thereby increasing the permeability of cell membranes and causing leakage of cell contents. In fact, the main antibacterial effects of daptomycin are the promotion of permeability and depolarisation of bacterial cell membranes (Taylor and Palmer, 2016). Iron is a key nutrient for almost all life forms, including bacteria, and is an important cofactor for many enzymes. It is an essential micronutrient for bacteria, has a relatively optimal redox chemistry, is involved in DNA synthesis, electron transport and energy metabolism, and is also a key determinant of bacterial virulence (Soares and Weiss, 2015). The imbalance in iron homeostasis within MRSA caused by Cu@Fe NPs may affect bacterial nutrient uptake and contribute to the change in bacteria from the sequestered phase to the planktonic phase, thus affecting bacterial survival. In addition, staphylococci can act as pathogens in part because of their ability to mitigate endogenous and exogenous oxidative and nitrosative stress, and both staphylococci and antioxidant enzymes can detoxify ROS and nitrogen intermediates (Gaupp et al., 2012; Cheung et al., 2021). ROS, especially OH-, cannot be scavenged by the corresponding enzyme systems and mainly reacts through DNA oxidation that indices oxidative damage and destroy the integrity of cells, ultimately leading to cell death and disrupting biological systems (Kim et al., 2019). The increase in excess exogenous ROS is also a common target of clinically used antibiotics, such as β-lactams, aminoglycosides, and quinolones, as inhibitors of bacterial growth. Thus, all three changes induced by Cu@Fe NPs within MRSA cells ultimately contribute to bacterial killing. Finally, the antibacterial effect of Cu@Fe NPs was validated *in vivo* using MRSA-induced systemic infection mouse models.

In summary, our novel Cu@Fe NPs with excellent anti-MRSA activity, effectively attenuate the progression of antimicrobial drug resistance. As an antibacterial strategy capable of reducing the likelihood of bacterial drug resistance, the multiple unique antibacterial modes exhibited by Cu@Fe NPs allow us to further explore the mechanism of action in the treatment of multidrug-resistant bacterial infections. Considering that the molecular targets of Cu@Fe NPs do not overlap with those of conventional antibiotics, based on the properties of NPs, if antibiotics are adsorbed on the surface of nanocarriers, the synergistic effect of both nanoparticles can improve the ability of loaded drugs to pass through cell membranes and the uptake of antibiotics, which ultimately leads to an increase in antibacterial activity (Azzopardi et al., 2013; Wong et al., 2013; Jelinkova et al., 2019). It has been reported that NPs as drug carriers can effectively load the first-line chemotherapeutic drug paclitaxel and improve the chemotherapeutic efficacy of paclitaxel in the treatment of cancers such as ovarian cancer (Wang et al., 2020; Wu et al., 2021). Therefore, Cu@Fe NPs in combination with antibiotic therapy is also a new avenue to be considered, which awaits further investigation. Overall, Cu@Fe NPs may serve as a new approach for the treatment of MRSA infections, and our findings will provide favorable theoretical support and experimental basis for this purpose.

## Materials and methods

### Bacterial strains

All bacteria used in this study were ATCC standard strains: MRSA (ATCC 337371), MSSA (ATCC 29213), and VISA (ATCC 311696).

### Synthesis of Cu@Fe NPs

CuCl_2_, FeCl_2_, and FeCl_3_ were dissolved in 100 mL water to the final concentrations of 0.1, 0.1, and 0.4 mM, respectively. Then, 500 mg of polyvinylpyrrolidone was added and mixed well to obtain a copper-iron precursor solution with a Cu^2+^:Fe^2+^:Fe^3+^ molar ratio of 1:1:4. NaOH was dissolved in a three-necked round-bottom flask containing 100 mL of deionized water to a concentration of 1.6 mM. After that, 500 mg of polyvinylpyrrolidone was added, and kept at 800–1,200 rpm to mix well on a hot oil bath. The NaOH solution was placed under the condenser tube. When the temperature of the NaOH solution reached 85°C–90°C, the copper-iron precursor solution was added dropwise at a rate of 3.5 mL/min using a syringe. After 30 min of reaction, the product was removed and centrifuged at 5,000 rpm. Thereafter, the product was successively washed with 5 mL water, 5 mL ethanol, 5 mL acetone, and 5 mL water by centrifugation. Finally, the washed product was dried in a vacuum oven for 24 h, 25°C and obtained as copper-iron oxide containing Cu_0.5_Fe^(II)^_0.5_Fe^(III)^_2_O_4_ (designated as Cu@Fe NPs). Cu@Fe NPs stored in a refrigerator at 5°C and sheltered from light for later use.

### MIC determination

The MIC was determined using the broth microdilution method according to the CLSI Clinical and Laboratory Standards Institute guidelines. Three replicate wells were made in a 96-well plate, and different concentrations of Cu@Fe NPs (0–128 μg/mL) were added to MRSA, MSSA and VISA bacterial solutions at a cell density of 5 × 10^5^ CFU. The 96-well plates were incubated at 37°C for 24 h, and the MIC value was determined as the lowest drug concentration at which no bacterial growth was observed by the naked eye.

### Bacterial resistance testing

The MIC values of SA and MRSA were determined as described above. After collection at doses second only to the MIC values, the bacteria were cultured and treated with a given dose of Cu@Fe NPs. The new MIC values were then calculated. Bacterial resistance assays were terminated after 56 replicate passages for MRSA and 40 replicate passages for SA, and MICs for different generations were recorded as MIC*n* (*n*, representing the number of generations). Ciprofloxacin and vancomycin were used as control drugs, and vancomycin was used as a positive control drug for MRSA. All measurements were repeated three times.

### Time-kill experiment

MRSA in the exponential growth phase was treated with different concentrations (0, 1, 2, 4, and 8 μg/mL) of Cu@Fe NPs at 37°C. At selected time points (3, 6, 9, 12, and 24 h), the bacterial suspensions were incubated overnight at 37°C by gradient dilution on tryptic soy agar plates. Finally, the CFU was used to calculate the number of viable bacteria.

### Haemolytic activity assay

Cu@Fe NPs (25–250 μg/mL) were added to 1 × 10^7^ RBC/mL C57BL/6 mouse erythrocyte suspension. PBS-containing solutions and Triton X-100 (1% v/v) were used as the negative and positive controls, respectively. After incubation for 1 h at 37°C, the OD was measured at 540 nm using a microplate reader (Biotek, Eon-Epoch 18,001,191). The percentage hemolysis was calculated as follows: Haemolysis (%) = [absorbance of sample − absorbance of blank] / absorbance of positive control] × 100. The concentration of the drug required to lyse 50% of the red blood cells (HC50) was used to calculate the 50% haemolytic value.

### Morphological observation *via* scanning electron microscopy

The morphology of MRSA was observed by scanning electron microscopy (SEM). MRSA (1 × 10^9^ CFU) was treated with Cu@Fe NPs and vancomycin (both at an equivalent concentration of 3.84 μg/mL) for 2 h. The bacterial solution was collected and centrifuged at 1320 g for 6 min at 4°C. Bacteria were fixed in 4% paraformaldehyde for 30 min. After fixation, the samples were centrifuged to discard paraformaldehyde, and then dehydrated with gradients of 25, 50, 75, and 100% ethanol solutions for 15 min in each step. The samples were directly coated with gold, without drying. Images were acquired using SEM (TESCAN, VFGA3LMu). Scale bar, 5 μm (200×). In addition, the morphology of mRBCs was observed by SEM. Cu@Fe NPs (3.84 μg/mL) were added to C57BL/6 mouse erythrocyte suspension diluted to a final concentration of 1 × 10^6^ RBC/mL in PBS and incubated at 37°C for 1 h. The mixture was then centrifuged at 1320 g for 6 min. The cells were resuspended twice in 1 mL PBS at 4°C and fixed in PBS containing 2.5% glutaraldehyde. After washing with PBS for three times, the erythrocytes were fixed with 1% OsO_4_-containing PBS for 1 h and dehydrated with a gradient of PBS and alcohol. The samples were air-dried and photographed with SEM. Scale bar, 10 μm (200×).

### HSF-1 cell viability assay

The effect of Cu Fe@NPs on the viability of human skin fibroblast (HSF-1) cells was assessed using the Cell Counting Kit-8 (GLPBIO, GK10001). The cells (5 × 10^3^ cells/well) were inoculated into 96-well plates, cultured for 24 h, and then treated with different concentrations (0–48 μg/mL) of Cu Fe@NPs. After 24 h of treatment, 10 μL CCK8 was added and incubated for 1–2 h at 37°C in the dark. The absorbance was measured using a multimode plate reader (Perkin Elmer, Victor Nivo 5S) at 450 nm. The lethal concentration (IC50) of Cu@Fe NPs in 50% cells was calculated as follows: Cell viability (%) = [absorbance of treatment − absorbance of blank] / [absorbance of control − absorbance of blank] × 100%.

### Cell membrane permeability assay

Bacterial membrane permeability was determined by fluorescence microscopy and spectrophotometry. The results of quantitative PI uptake by Cu@Fe NPs after 2 h of continuous MRSA action were compared with those of cells treated with vancomycin at the same concentration (3.84 μg/mL). MRSA (1 × 10^9^ CFU) was incubated with Cu@Fe NPs and vancomycin at 37°C with stirring at 180 rpm for 2 h. PI was added at a final concentration of 20 μM, and the mixed solutions were incubated at 37°C for 10 min in the dark. Each mixture was taken and fixed on a slide for observation through a fluorescence microscope (Zeiss, Axio Imager. A2), and then photographed through a digital camera (Zeiss, AxioCam503). Magnification, 1,000×. Scale bar, 10 μm. For spectrophotometric analysis, MRSA (1 × 10^9^ CFU) was incubated with Cu@Fe NPs and vancomycin (both at a concentration of 3.84 μg/mL) at 37°C with stirring at 180 rpm for 30 and 60 min. PI was added and incubated for 10 min at 37°C in the dark, and the relative fluorescence units (RFU) were measured using a fluorescent microplate reader at 535/ 617 (excitation/emission).

### Biofilm inhibition experiment

The MRSA broth was diluted to a final cell density of 1 × 10^6^ CFU and transferred to 96-well plates containing Cu@Fe NPs at concentrations of 1.2, 2.4, 4.8, and 7.2 μg/mL. Incubation was carried out at 37°C for 24 h to form biofilms. After incubation, the biofilm was gently washed with PBS to maintain the integrity of the biofilm. The biofilm was scraped off with a cytospatula and the OD was measured at 600 nm on a microplate reader. The results were expressed as a percentage change relative to the control (no nanoparticles). The biofilm was gently disrupted and spread on TSA after serial dilutions of each suspension, and the number of live bacteria in the sample was counted. The results were expressed as a percentage change relative to the control.

### Biofilm disruption experiment

Overnight TSB cultures of MRSA were diluted with 0.5% glucose to a final cell density of 2 × 10^6^ CFU and transferred to 96-well plates. After incubation at 37°C for 24 h to form biofilms, TSB medium containing varying concentrations (4–256 μg/mL) of Cu@Fe NPs was added to the biofilms and incubated for a further 24 h. The biofilms were scraped off with a cell scraper, and then suspended with PBS solution. The OD was measured at 600 nm using a microplate reader to determine the quality and quantity of the biofilms. All data were expressed as a percentage change relative to the control.

### Biofilm staining experiment

The MRSA broth was diluted to a final cell density of 1 × 10^6^ CFU, and transferred to 6-well plates containing Cu@Fe NPs at 2.4, 4.8, and 7.2 μg/mL. After incubation at 37°C for 24 h to allow biofilm formation, the biofilms were gently washed to maintain biofilm integrity. A solution containing SYTO9 and PI (Thermo Fisher, Scientific LIVE/DEAD BacLight Bacterial Viability Kit L13152) was then added to the biofilm-mounted plates, which were incubated at room temperature in the dark. Magnification, 200×. Scale bar, 5 μm. Each sample well was then washed with PBS and fluorescence images were obtained using an inverted fluorescence microscope (Leica Dmi8-462,940).

### Measurement of intracellular iron

Bacterial intracellular iron was measured using an iron assay kit (ab83366, Abcam). MRSA at a cell density of 2 × 10^8^ CFU was prepared in TSB medium, and the solution was treated with different concentrations (0.5, 1 and 2 μg/mL) of Cu@Fe NPs at 37°C with stirring at 180 rpm for 2 h. After centrifugation at 3,000 rpm for 6 min at 4°C, the supernatant was discarded and washed twice with PBS. Subsequently, the solution was transferred to a 96-well plate and an appropriate volume of iron assay buffer and 5 μL of iron reducing agent was added to each well. Mix and incubate at 37°C for 30 min in the dark. Then, 100 μL iron probe was added to each well containing the iron standard and test samples. The mixture was incubated for 60 min at 37°C in the dark. The OD of each well was measured at 593 nm using a microplate reader and the total iron content (nM) of the samples was calculated from the standard curve. The number of viable bacteria in the sample was determined by agar plate counting method. The total iron content was normalized by the number of viable bacteria (CFU/mL).

### Iron rescue experiment

The MRSA suspension (1 × 10^6^ CFU) was treated with a mixture of Cu@Fe NPs and iron citrate at a concentration of 7.5 μg/mL for 24 h. Different ratios of Cu@Fe NPs:Fe^3+^ were 1:0.01, 1:0.05 and 1:0.1. The number of viable bacteria in each sample was determined by agar plate count.

### Measurement of intracellular ROS

Intracellular levels of ROS in the bacteria were determined using a bacterial ROS assay kit (Biorab, Beijing, HR9066). The MRSA broth at a cell density of 2 × 10^8^ CFU was incubated with different concentrations (0.5 and 1 μg/mL) of Cu@Fe NPs and ROS probes at 37°C for 15, 30, and 60 min. The RFU values were measured using a fluorescent microplate reader at 495/529 (excitation/emission).

### ROS scavenging experiment

MRSA at a cell density of 1 × 10^6^ CFU was treated with different concentrations of Cu@Fe NPs and incubated at 37°C for 24 h at 180 rpm with and without the addition of 500 μL SOD (250 U/mL; Medicalsystem Biotechnology, JZ439). There are several ways to determine bacterial growth, among them, CFU and OD600 values have been widely used in different infection models (Heller and Spence, 2019; Kawakami and Tagami, 2021). The relative bacterial number was assessed by measuring the absorbance of the samples using a microplate reader at 600 nm.

### Establishment of animal models

Adult male C57BL/6 mice (6–8 weeks old) were provided by the Hunan Branch of the Experimental Animal Center of the Chinese Academy of Sciences. All procedures were performed in accordance with the Guide for the Care and Use of Laboratory Animals, issued by the Ministry of Health of the People’s Republic of China. The mice were allowed to acclimatize to their new environment for one week, and were housed in the SPF facility at the Gannan Medical University Experimental Animal Center in Ganzhou, Jiangxi Province, China. The mice were fed a standard diet and sterile water. The feeding conditions were as follows: temperature (22°C–26°C); humidity (50 ± 5%); and light/dark cycle of 12 h. This study was approved by the ethics committee of Gannan Medical University.

### MRSA systemic infection model

The systemic and localized MRSA infection models were constructed as previously described (Yu et al., 2020; Liu et al., 2021). Briefly, 100 μL MRSA solution (1 × 10^8^ CFU) was injected intraperitoneally into C57BL/6 male mice on day 0 and randomly divided into three groups: control (*n* = 18), vancomycin (*n* = 12), and Cu@Fe NPs (*n* = 14). On day 1 post-infection, the mice were intraperitoneally injected with a single dose of sterile saline, vancomycin (7.8 mg/kg), and Cu@Fe NPs (7.8 mg/kg). The mice were executed on day 2 after infection, and their organs such as the liver, spleen, kidney and lung were immediately collected and homogenized for bacterial CFU counting.

### MRSA localized skin wound infection model

The dorsal skin of mice was debrided on day 0 of infection using an animal shaver and debridement cream, and full-thickness circular wounds were created on the dorsal skin of C57BL/6 male mice using a 6-mm sterile biopsy punch. Then, 25 μL MRSA solution (1 × 10^7^ CFU) was inoculated onto the injured skin and randomly divided into three groups (n = 4/group): control group, vancomycin group, and Cu@Fe NPs group. The skin wounds were covered with a clear, semi-permeable Tegaderm-3 M dressing. On day 1 post-infection, 25 μL of sterile solvent solution, vancomycin solution (0.4 mg/kg), and Cu@Fe NPs solution (0.4 mg/kg) were administered topically to the infected area of the wounds. Animals were executed on day 2 post-infection and the skin flap around the circular wound was immediately excised and homogenized for bacterial CFU counts.

### Statistical analysis

Statistical tests were performed with Graphpad prism 8.0. Data are expressed as mean ± standard error (SEM). Significant differences between groups were compared by Student t-test or analysis of variance (ANOVA). *p < 0.05 was considered statistically significant.

## Data availability statement

The raw data supporting the conclusions of this article will be made available by the authors, without undue reservation.

## Ethics statement

The animal study was reviewed and approved by this study was approved by the Ethics Committee of Gannan Medical University.

## Author contributions

YT, JH, and JY: Conceptualization and methods. GC: Synthesis of Cu@Fe NPs. TZ and DL: Data analysis. FH and YY: Investigation. JX and YL: Validation. FY: Supervision. FH: Writing-original draft preparation. JY and JH: Writing-review and editing. YT: Funding acquisition. JY, FH, and GC contributed equally to this work. All authors contributed to the article and approved the submitted version.

## Funding

This study was supported by the Gannan Medical College Innovation Team Project (TD201703).

## Conflict of interest

The authors declare that the research was conducted in the absence of any commercial or financial relationships that could be construed as a potential conflict of interest.

## Publisher’s note

All claims expressed in this article are solely those of the authors and do not necessarily represent those of their affiliated organizations, or those of the publisher, the editors and the reviewers. Any product that may be evaluated in this article, or claim that may be made by its manufacturer, is not guaranteed or endorsed by the publisher.
